# An Overview of Pathogen Recognition Receptors for Innate Immunity in Dental Pulp

**DOI:** 10.1155/2015/794143

**Published:** 2015-10-20

**Authors:** Ji-Hyun Jang, Hee Woong Shin, Jung Min Lee, Hyeon-Woo Lee, Eun-Cheol Kim, Sang Hyuk Park

**Affiliations:** ^1^Department of Conservative Dentistry, Kyung Hee University Dental Hospital at Gangdong, Seoul, Republic of Korea; ^2^School of Dentistry, University of Western Australia, Nedlands, WA, Australia; ^3^Department of Conservative Dentistry, Graduate School, Kyung Hee University, Seoul, Republic of Korea; ^4^Department of Pharmacology, School of Dentistry, Kyung Hee University, Seoul, Republic of Korea; ^5^Oral Biology Research Institute, School of Dentistry, Kyung Hee University, Seoul, Republic of Korea; ^6^Department of Maxillofacial Tissue Regeneration, School of Dentistry and Institute of Oral Biology, Kyung Hee University, Seoul, Republic of Korea; ^7^Department of Conservative Dentistry, School of Dentistry, Kyung Hee University, 1 Hoegidong, Dongdaemoongu, Seoul 130-701, Republic of Korea

## Abstract

Pathogen recognition receptors (PRRs) are a class of germ line-encoded receptors that recognize pathogen-associated molecular patterns (PAMPs). The activation of PRRs is crucial for the initiation of innate immunity, which plays a key role in first-line defense until more specific adaptive immunity is developed. PRRs differ in the signaling cascades and host responses activated by their engagement and in their tissue distribution. Currently identified PRR families are the Toll-like receptors (TLRs), the C-type lectin receptors (CLRs), the nucleotide-binding oligomerization domain-like receptors (NLRs), the retinoic acid-inducible gene-I-like receptors (RLRs), and the AIM2-like receptor (ALR). The environment of the dental pulp is substantially different from that of other tissues of the body. Dental pulp resides in a low compliance root canal system that limits the expansion of pulpal tissues during inflammatory processes. An understanding of the PRRs in dental pulp is important for immunomodulation and hence for developing therapeutic targets in the field of endodontics. Here we comprehensively review recent finding on the PRRs and the mechanisms by which innate immunity is activated. We focus on the PRRs expressed on dental pulp and periapical tissues and their role in dental pulp inflammation.

## 1. Introduction

The innate immune response is the first line of defense against infectious diseases and tissue damage. Macrophages and dendritic cells (DCs), as well as some nonprofessional cells such as epithelial cells, endothelial cells, and fibroblasts, play major roles in pathogen recognition during the innate immune response [1]. Cells of the host recognize structures called pathogen-associated molecular patterns (PAMPs) via germ line-encoded pattern recognition receptors (PRRs) present in their extracellular milieu and endosomal compartments [2]. Currently, PRR families are divided into transmembrane receptors and those that reside in intracellular compartments. The former include the Toll-like receptors (TLRs) and C-type lectin receptors (CLRs), and the latter, the nucleotide-binding oligomerization domain- (NOD-) like receptors (NLRs), retinoic acid-inducible gene- (RIG-) I-like receptors (RLRs), and AIM2-like receptor (ALR) [1, 3, 4]. PAMP recognition by PRRs is influenced by both the responding cell and the invading microorganism. The signal transduction pathways that are activated via PRRs converge on a common set of signaling modules including nuclear factor- (NF-) *κ*B, activator protein-1 (AP-1), and mitogen-activated protein kinase (MAPK). These modules drive the production of proinflammatory cytokines/chemokines such as interleukin- (IL-) 1, tumor necrosis factor- (TNF-) *α*, and IL-6 [1, 2, 5]. Cytokines are multifunctional proteins that regulate osteoclast formation and hence bone resorption, modify vascular endothelial permeability, and recruit immune cells to inflamed tissue [2, 6].

Over the past decade there have been rapid advances in understanding innate immunity, particularly with regard to the mechanisms by which microbes are recognized and how the signaling molecules respond to them. Accumulating evidence of a relationship between bacterial recognition systems and oral disease has focused attention on the role of dental pulp tissues and their associated pathogens in innate immunity. In this review, we comprehensively review recent finding on the PRRs and the mechanisms by which innate immunity is activated. We will describe recent findings concerning the receptors for innate immunity in dental pulp.

## 2. Dental Pathogens and Innate Immunity 

Teeth have unique structural features not found in any other tissue of the body. The hard tissues, enamel and dentin, make up the rigid external surface of the tooth, while its internal milieu is composed of soft tissue called “pulp.” The pulp responds to external pathologic stimuli such as bacterial ingress and trauma, as well as thermal and chemical irritation during dental operations, all of which may induce inflammation [7, 8]. Pulp resides in a low compliance root canal system that limits the expansion of inflamed pulpal tissue that is invaded by inflammatory cells and whose blood vessels dilate [9, 10].

In the interface layer between dentin and pulp, there is a thin border which consists of odontoblasts and cells in a subodontoblastic layer [11]. Odontoblasts, the most highly differentiated cells of the pulp, are postmitotic neural crest-derived cells whose primary function is to elaborate dentin [12]. In response to irritation by cariogenic bacteria, odontoblasts produce tertiary dentin [13]. This has been classified as either reactionary or reparative, to distinguish between the events taking place in response to weaker versus stronger stimuli, and results from upregulation of the secretory activity of existing odontoblasts [12]. If the pulp is exposed, odontoblasts in the dentin pulp can no longer perform reparative processes. In the pulp, fibroblasts are the most numerous connective tissue cells, and they synthesize and maintain the connective tissue matrix [11]. Cariogenic bacteria trigger inflammatory and immune events in the underlying dental pulp via diffusion of their by-product into dentin tubules. If the bacteria are not eliminated, lesions progress to pulp inflammation and are followed by infection of the root canal system and periapical tissues and eventually by periapical disease [13].

Dental pathogens gain access to the dental pulp through the carious process and/or iatrogenic damage from dental treatments including cavity preparation and the use of cytotoxic dental materials. Dental caries harbour a wide range of bacteria, viruses, fungi, and protozoa within the mineralized tissues and canals of the root [14, 15]. When enamel structure is destructed, the dentin exposed to the oral microflora is degraded by Gram-positive bacteria, such as streptococci, lactobacilli, and actinomyces. Once bacterial infection due to dental caries progresses to the dentin-pulp interface, microflora is changed drastically. It is characterized by a reduction of Gram-positive aerobic bacteria with an increase of Gram-negative anaerobic bacteria, and initial pulpal immune response is activated [16–18]. It releases various bacterial toxins such as lipopolysaccharide (LPS), lipoteichoic acid (LTA), and some noxious metabolic by-products that will induce pulpal and periapical inflammatory reaction, followed by the results in irreversible pulpitis, pulp necrosis, and periapical disease [19–21].

It has been estimated that the human oral cavity is colonized by over 700 different species of bacteria [22]. The surface of the tooth accumulates bacteria in biofilms. The main bacterial species include streptococci (such as* Streptococcus mutans*) and* Actinomyces* spp. [23, 24]. The gingival crevices contain Gram-negative anaerobes such as* Porphyromonas gingivalis*, many of which are believed to be important in the development of periodontal disease [22, 24].* Candida albicans* is the most common fungus present in the oral cavity, especially in the root canals [24, 25]. Protozoa, such as* Entamoeba gingivalis,* and viruses, including herpes virus and cytomegalovirus, are often present in the mouth [25]. Bacteraemia, endocarditis, atherosclerosis, and other cardiovascular diseases have been linked to oral pathogens that gain systemic access [26].

The innate immune system is the first line of pulp defense, triggered by pathogen recognition in a cell-autonomous manner [27]. The inflammatory process is mediated by PRRs which are expressed by various immune and nonimmune cells [2]. Innate immunity depends on the release of local mediators and phagocytic cells such as macrophages, monocytes, neutrophils, and DCs, whereas adaptive immunity uses antigen-specific T and B cells [28]. Phagocytic cells form an important part of the innate immune response. These cells directly remove pathogens that they encounter by phagocytosis but also release inflammatory cytokines and chemokines, which recruit other immune cells to the site of infection [29]. The expression of PRRs on host cells allows them to recognize specific pathogens, hence conferring a degree of specificity to the innate immune system. The DCs also express PRRs and act as cellular messenger by binding antigens and migrating to the lymph nodes where they activate the adaptive immune system [30]. The activation of PRRs can cause apoptosis and inflammation as well as stimulating adaptive immunity [1, 2, 31].

## 3. Pathogen Recognition Receptors

The defense mechanisms of the dental pulp comprise both innate and adaptive immunity. A critical first step in initiating an innate immune response to infection is the sensing of the pathogens by host cells. This is mediated by the recognition of specific microbial molecules by a limited array of dedicated host receptors. The microbial ligands, corresponding to essential components of the pathogen, are PAMPs and their cognate PRRs. As we described previously, PRRs are classified into five main families: TLRs and CLRs, transmembrane proteins found in the plasma membrane, and RLR, ALR, and the NLR proteins located in intracellular compartment [3]. Here, we describe each of the PRR families and review recent findings on PRRs.

### 3.1. Toll-Like Receptors

Toll-like receptors (TLRs) are a class of proteins that play a key role in the innate immune system and received their name from their similarity to the protein encoded by the* toll* gene in* Drosophila* [56]. Drosophila Toll is involved in both embryonic development and the immune response to fungi [56, 57]. TLRs are a family of receptors with conserved architecture consisting of leucine-rich repeat- (LRR-) containing ectodomains and intracellular Toll-interleukin-1 receptor (TIR) signaling domains. The TLR ectodomains contain numerous LRRs, each repeat consisting of a 24-residue motif [1, 58].

The TLRs include TLR1–TLR10 and TLR11–TLR13, though the latter three are not found in humans. There are 10 TLR family members, TLR1–TLR10, in humans and 12, TLR1–TLR9 and TLR11–TLR13, in mice [59]. TLRs are able to recognize a variety of PAMPs including lipoproteins and di- and triacyl lipopeptides (TLR2/1 and TLR2/6), peptidoglycan, lipoteichoic acid, fungal zymosan (TLR2), double-stranded RNA (TLR3), flagellin (TLR5), unmethylated CpG DNA (TLR9), and a variety of synthetic molecules such as imidazoquinolines and guanosine analogues (TLR8). These molecules are recognized by individual TLRs in combination with coreceptors, or by TLR heterodimers [59]. Different TLRs appear to play crucial roles in the initiation of immune responses by recognizing different PAMPs. Odontoblasts constitutively express the PRRs TLR1–TLR6 and TLR9 genes [60].

TLR signaling is triggered by the ectodomain-mediated dimerization of TLRs. Its signaling involves two distinct signaling pathways: the myeloid differentiation factor 88- (MyD88-) dependent and TIR (Toll-interleukin receptor) domain containing adapter-inducing interferon- (IFN-) *β*- (TRIF-) dependent pathway. Those signaling pathways lead to activation of NF-*κ*B protein, which is a cytoplasmic transcription factor that initiates transcription of a wide range of genes involved in the inflammatory response including cytokines, chemokines, and immunoreceptors [58]. MyD88 is utilized by all TLRs with the exception of TLR3 and drives NF-*κ*B and MAPK activation to control inflammation [61]. TLR3 and TLR4 utilize the TRIF-dependent pathway, which is triggered by dsRNA and LPS, respectively [53, 62]. TRIF is also known as TIR domain containing adapter molecule (TICAM) 1, and it selectively recruited to their respective TLRs, eliciting appropriate responses depending on the type of PAMP [2].

TLR2 has also been designated CD 282. It is a surface membrane receptor that recognizes foreign substances and signals to cells of the immune system [63]. LTA stimulates the activation of odontoblasts, which is followed by expression of its receptor, TLR2 [32]. Murine pulp fibroblasts and odontoblasts have been shown to express TLR2 [39]. TLR2 is involved in the recognition and development of immunological responses against Gram-positive bacteria; it has a major role in the detection of peptidoglycan, lipoprotein, and LTA [39]. When TLR2 interacts with LTA, TLR2 gene expression in the cell membrane increases, NF-*κ*B translocates to the nucleus inducing the production of chemokines (via the chemokine genes* CCL2* and* CXCL8*), and immature DCs are recruited by upregulation of NOD2 expression [19, 35]. TLR2 is closely related to TLR1 and TLR6 with which it forms heterodimers that recognize bacterial lipoproteins and lipopeptides [34, 64]. TLR2 and TLR6 are required for responses to the diacyl lipoprotein from* Mycoplasma fermentans* [65]. TLR2 is expressed by neutrophils, mast cells, monocytes and macrophages, T cells, and B cells [45]. Macrophages and lymphocytes are the most prevalent cells in periapical infiltrates and produce IL-1, TNF, nitric oxide (NO), and reactive oxygen species (ROS). During the development of periapical lesions, macrophages and other innate immune response cells recognize bacterial constituents via specific receptors and initiate the inflammatory cascade [35, 45].

TLR4 is crucial for the detection of LPS, which is present in the cell wall of Gram-negative bacteria [66]. TLR4 is also expressed in the odontoblastic layer and pulp tissues [33]. Upon activation, TLR4 induces the production of proinflammatory cytokines, and cytokine expression is associated with bone resorption and tissue breakdown in endodontic periapical lesions [34, 35]. Mutoh et al. investigated the expression of TLR2 and TLR4 in inflamed dental pulp. They showed that TLR2 was strongly expressed on macrophages and DCs. TLR4-positive cells were also detected in the pulp, but the number of cells expressing it was much lower than in the case of TLR 2 [34].

The TLR5 ligand is flagellin, the major component of the bacterial flagellum and the structure responsible for motility in a wide variety of bacterial species [67]. A range of flagellated bacteria, but not aflagellate strains, activate TLR5 [68]. Interestingly, certain species of bacteria including* Campylobacter jejuni*,* Helicobacter pylori*, and* Bartonella bacilliformis* possess a divergent flagellin, which is not recognized by TLR5 due to amino acid mutations at residues 89–96 [68]. These amino acids correspond to a region previously defined as important for TLR5 activation. They are critical for flagellar filament formation and motility in other species, which explains their extensive conservation. Compensatory mutations in other regions of the protein are able to restore motility in these divergent species [68].

TLR3, TLR7, TLR8, and TLR9 are different from other TLRs in a view point in which they are not expressed in surface but localized on cytoplasmic vesicles such as endosomes. They are involved in the recognition of nucleic acids, with TLR3, TLR7, and TLR8 detecting double- and single-stranded RNA, respectively, and TLR9 detecting unmethylated CpG DNA [63]. In recognizing double-stranded RNA, TLR3 acts as a viral receptor, as dsRNA is present in certain viruses. Examination of the crystal structure of the human TLR3 ectodomain, in combination with mutational analysis, has identified two highly conserved residues critical for ligand binding and TLR3 activation within LRR 20 of the ectodomain [63].

TLR7 and TLR8 are structurally highly conserved proteins that interact with some of the same ligands. They are predicted to recognize the nucleic acid structures of viruses [69]. TLR7 is required for the normal IFN-*α* response to influenza in murine DCs [63]. In contrast, human TLR8 responds to these oligonucleotides independently of TLR7, pointing to species-specific differences between human and mouse. Binding of the single-stranded RNA virus, vesicular stomatitis virus (VSV), to mouse TLR9 also induces secretion of IFN-*α*. This process requires the acidification of lysosomes and is inhibited by chloroquine, implying compartmentalization of the TLR7 response, possibly as a way of distinguishing self-RNA from non-self-RNA [69]. A number of other compounds activate TLR7, including various synthetic analogues of guanine, such as imiquimod, resiquimod, and loxoribine. Most are specific to TLR7, although resiquimod is also able to activate TLR8 [70].  Table 1 presents the overview of the TLRs and their PAMPs in human innate immunity.

### 3.2. C-Type Lectin Receptors

CLRs possess a transmembrane PRR with a carbohydrate-binding domain. CLRs recognize carbohydrates on pathogens and are mainly expressed by monocytes, macrophages, and DCs [3]. Pathogen recognition by CLRs leads to pathogen internalization and degradation and subsequent antigen presentation. CLRs recognize mannose, fucose, and glucan carbohydrate structures present in bacterial, viral, and fungal components. They are crucial for controlling both innate and adaptive immune responses. Some CLRs induce signaling pathways that modulate TLR-induced gene expression [71]. Mincle, a C-type lectin, detects infection by fungi; and it is sensed on both monocytes and neutrophils as well as on macrophages differentiated* in vitro* [72]. Inflammatory responses are crucial in innate immunity against infectious disease, but the factors that determine the dominant cellular component have not been identified. The association between these patterns and phagocyte function is currently being investigated.

### 3.3. Nod-Like Receptors

The nucleotide-binding oligomerization domain receptors, in short NLRs, are intracellular sensors of PAMPs that enter the cell via phagocytosis or pores and of danger-associated molecular patterns (DAMPs) that are associated with cell stress. They are pattern recognition receptors and play key roles in regulating the innate immune response. NLRs can cooperate with TLRs and regulate inflammatory and apoptotic responses. They are found in lymphocytes, macrophages, and DCs and also in nonimmune cells, for example, in epithelia. NLRs are characterized by their cytoplasmic location and the possession of a nucleotide-binding domain (NBD), which is also emerging as an important component of the innate immune response [4]. NLRs constitute a large family of intracellular PRRs, several of which—such as NOD1, NOD2, and NALP3 (which are characterized by NACHT [neuronal apoptosis inhibitory protein (NAIP), CIITA, HET-E, and TP-1] domain, LRR (leucine-rich repeat) domain, and PYD (pyrin domain) containing protein 3) [2]. NALP3 is also known as NLRP3 (NLR family which has pyrin domain containing protein 3).

NOD1 and NOD2 recognize peptidoglycan components common to both Gram-positive and Gram-negative bacteria. Both proteins drive activation of MAPK and NF-*κ*B pathways, leading to proinflammatory cytokine production [2, 73]. Girardin et al. proved that human NOD1 specifically recognized a unique muropeptide motif found in Gram-negative bacterial peptidoglycan, resulting in activation of the NF-*κ*B responses [73]. NOD2 also responds to bacterial peptidoglycan and mediates the response to Gram-positive peptidoglycan, such as that from* Bacillus subtilis*. Again, digestion and fractionation of peptidoglycan identified specific fractions that simulated NOD2 [74].

NALP is a type of NOD-like receptor. NOD1 and NOD2 recognize intracellular bacterial cell products, but NALP3 responds to multiple stimuli to form a multiprotein complex termed the NALP3 inflammasome [2]. It is thought that NALP proteins sense inherent danger and link this with microbial products, creating a response mediated by the inflammasome that includes K^+^ efflux and caspase-1 activation [75]. NALP3 is required for the secretion of IL-1*β* and IL-18 that occurs when both bone marrow and peritoneal macrophages are stimulated with TLR7 ligands. NALP3 is required for caspase-1 activation, IL-1*β* secretion, and cell death when macrophages are infected with the Gram-positive bacteria* Staphylococcus aureus* and* Listeria monocytogenes*, suggesting that it is involved in the response to specific bacterial pathogens, and may be limited to Gram-positive species [76].  Table 1 presents the overview of the NLRs and their PAMPs in human innate immunity.

### 3.4. RIG-Like Receptors (RLRs)

Virus infection of mammalian cells triggers innate immune defenses through the PRRs for PAMPs within viral products that engage the intracellular signaling pathways to initiate an antiviral response. Viral RNA is a potent inducer of this host response and is recognized by specific TLRs or by cytoplasmic RNA helicases [62]. RLRs are intracellular receptors for RNA viruses. The RLR family is composed of at least 3 members: RIG-I, melanoma differentiation factor-5 (MDA5), and laboratory of genetics and physiology-2 (LGP-2). Studies of human cells defective in RIG-I signaling, or of cells from mice with a targeted deletion of RIG-I or MDA5, have revealed a remarkable degree of specificity of virus recognition between the individual helicases that could reflect differences in RNA binding and PRR function. Recognition by RLRs activates innate antiviral responses, mainly through the rapid induction of type I IFNs and inflammatory cytokines that limit viral replication and coordinate an antigen-specific, adaptive immune response [1, 2]. Each protein carries a helicase domain and a repression domain, and RIG-1 and MDA5 also possess two repeated N-terminal CARD domains. RIG-1 recognizes double-stranded RNA, activating IFN regulatory factor 3 (IRF3) and producing the key antiviral cytokines, type I IFNs [77].

**Figure 1:**
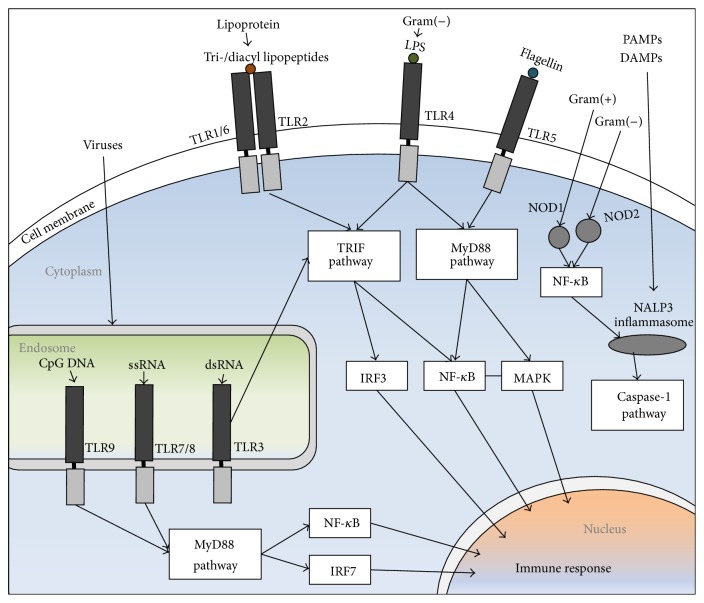
Schematic overview of TLR and NLR signaling pathways. PAMPs and DAMPs are recognized by PRRs. Heterodimer of TLR1/6+TLR2, TLR4, and endosomal TLR3 activate TRIF pathway, followed by induction of IRF and NF-*κ*B. TLR5 and endosomal TLR9 and TLR7 activate MyD88 pathway, followed by activation of MAPK, NF-*κ*B, and IRF7. NOD1 and NOD2 are cytoplasmic PRRs, and they trigger NF-*κ*B, and NALP3 inflammasome recruits and activates caspase-1 pathway. DAMP: damage-associated molecular patterns; IRF: IFN-regulatory factor; MAPK; mitogen-activated protein kinase; MyD88; myeloid differentiation primary-response gene 88; NF-*κ*B: nuclear factor-*κ*B; NALP3: NACHT, LRR, and pyrin domain containing protein 3; NOD: nucleotide-binding oligomerization domain; NLR: NOD-like receptor; PAMP: pathogen-associated molecular patterns; TLR: Toll-like receptor; TRIF: Toll/IL-1R (TIR) domain containing adaptor protein inducing IFN-*β*.

MDA5 and RIG-I exhibit limited homology, with 23% and 35% homology in their CARD and helicase domains, respectively. LGP2 lacks a CARD domain, and the helicase domain has 31% and 41% homology with RIG-I and MDA5, respectively [78]. Activation of RIG-I or MDA5 increases IFN-*β* secretion and activation of the IRF3 transcription factor, suggesting that these two RLR proteins activate the same signaling pathway [78]. LGP2 strongly inhibited the expression of an IFN-*β* reporter gene and impaired IRF3 dimerisation, indicating that it has a negative regulatory role. Both MDA5 and LGP2 bind to double-stranded RNA. MDA5 and RIG-I, but not LGP2, reduced viral yields following infection with EMCV and VSV. RIG-I and MDA5, therefore, seem to be important in the IFN response to certain viruses, which seems to be, at least partly, a response to RNA [12].  Figure 1 presents the schematic overview of NOD1, NOD2 and NALP signaling pathways.

## 4. Pathogen Recognition Receptors in Dental Pulp and Periapical Tissues

The past decade has seen a rapid development of researches on innate immunity and PRR in dental pulp and periapical tissues (Table 2). The pulp has many MHC class II positive cells such as odontoblasts, pulp fibroblasts, and dendritic, endothelial, and neural cells, which are the most active antigen presenting cells (APCs) initiating immune responses to dental pathogens [34].

As we described previously, immune cells infiltrate into the odontoblastic layer close to a lesion where dentin is being destroyed by cariogenic bacteria. Thus, odontoblasts are the first cells encountered by pathogens entering dental pulp. Odontoblasts express TLR1–TLR6 and TLR9 but not TLR7, TLR8, and TLR10 [32, 53]. They recognize bacterial products including triacetylated lipopeptides (TLR2/TLR1) [42], diacetylated lipopeptides (TLR2/TLR6) [32], viral RNA (TLR3, TLR7, TLR8, and TLR9) [53], LPS (TLR4) [33, 50], flagellin (TLR5) [32], and unmethylated CpG DNA (TLR9) [32, 47]. TLR2 activation by LTA induces the differential production of certain proinflammatory cytokines, and it increases the ability of odontoblasts to recognize and respond to a wide variety of bacterial and viral by-products [32, 39, 42].

Once pulp inflammation initiated, various proinflammatory mediators and cytokines are upregulated in dental pulp, especially pulp fibroblasts. Recent researches demonstrated that TLR2, TLR3, TLR4, and TLR5 [31, 39, 40] and NOD1 and NOD2 [16, 38] are expressed by human pulp fibroblasts. TLR2 and TLR4 are expressed in various inflammatory cells and odontoblasts in inflamed pulp tissue [34, 36]. Activation of TLR2, TLR3, and TLR4 by their specific ligands induces the production of proinflammatory and chemokine proteins such as CCL2, CCL5, CCL7, CXCL8, and CXCL10 [40, 79]. TLR2 acts synergistically with NOD2 to stimulate proinflammatory mediator production in human pulp fibroblasts [39]. It also synergizes with the inflammation mediator (histamine receptor 1); thus fibroblasts express functional receptors that recognize pathogens and are potential initiators of immune/inflammatory events in dental pulp [40].

TLR2 is involved in detecting the Gram-positive bacterial components that dominate the microflora of failed root canal treatments.* Enterococcus faecalis* can survive in harsh environment and is known as one of the major etiologic factors in various stages of persistent periapical disease.* E. faecalis*, a Gram-positive facultative anaerobic bacterium, possesses antigenic LTA and lipopeptide components. These activate the TLR2/TLR1 complex in human odontoblasts. Transcription of inflammatory cytokines IL-8 and TNF-*α* is also increased [19, 49]. Chlorhexidine reduces the ability of LTA antigen to be recognized by TLR2, and this decreases the production of TNF-*α*. Refractory periapical diseases contain large numbers of intraradicular Gram-positive bacteria [80].

TLR2 expression in various periapical lesions may play a role in the recognition of the atypical LPS of* P. gingivalis* [34]. T lymphocytes dominate the chronic periapical granuloma, and* in vitro* experiments showed that TLR2 is expressed in CD4+, CD3+, and CD14− T cells [81]. As mentioned above, TLR2 has an indirect role in adaptive immunity through the activation of APCs. However, its role extends to direct augmentation of antigen-specific Th1 responses. Sustained expression of TLR2 on memory T cells allows an immediate strong response on encountering a previously recognized pathogen [81]. Treg cells regulate and dampen Th cell-mediated immune reactions. Their activity may lead to various autoimmune diseases and inadequate development of an effective immune response during infection. Upon direct contact with bacterial ligands, TLR2 is expressed on Treg cells [82]. In periapical lesions, Treg cells produce TGF-*β*, which is responsible for inhibiting Th1-mediated cytokines. Exposure to TFG-*β* abolishes the TLR2-mediated responses of odontoblasts [37].

Odontoblasts and pulp fibroblasts express TLR4 in response to antigen challenge. In inflamed pulp model, TLR4 expression on pulp macrophage and dendritic-like cells was lower and slower compared to that of TLR2. Root canal pathogens stimulate TLR2 and TLR4, and they participate in the induction and progression of periapical lesion through NO and ROS production by activated macrophages [35]. TLR4 is involved in detecting the Gram-negative bacterial component LPS (lipid-A portion).* P. gingivalis* is often retrieved from infected root canal systems. The lipid-A subunit of LPS obtained from* P. gingivalis* has several different structures. Although TLR2 does not play an active role in the recognition of Gram-negative bacteria, heterogeneous LPS can activate host immune cells through a TLR2-dependent pathway [34, 83]. Botero et al. reported that LPS is associated with recognition of TLR2 and TLR4, and it induces vascular endothelial growth factor (VEGF) expression in dental pulp via MAPK activation [41]. TLR4 was detected in the early stage of pulp inflammation in experimentally inflamed pulps in mouse model [36].

NLRs share common features with TLRs in that ligand binding is mediated by LRR domain. Hirao et al. demonstrated that human pulp fibroblasts constitutively express intracellular NOD1 and NOD2 as well as TLR2 and TLR4, and each PRR-specific ligand was upregulated to produce various proinflammatory mediators, suggesting that NODs have a potent influence on proinflammatory responses in dental pulp [16]. NOD1 and NOD2 participate in the innate immune response through the NF-*κ*B pathway, and NOD1/NOD2 signaling has been reported to trigger IL-8 expression. NOD1 and NOD2 are expressed in normal dental pulp, and their expression is upregulated in inflammatory responses [6, 18, 38]. NOD2 participated in the odontoblast differentiation via downregulation of MAPKs and osteoclastogenesis by providing macrophage colony-stimulating factor (M-CSF) and receptor activator of NF-*κ*B ligand (RANKL) in the presence of muramyl dipeptide (MDP) [6, 43]. MDP also activates NOD2-specific si-RNA, followed by upregulation of the TLR2, TLR4, and NALP3 signaling pathways in dental pulp cells to trigger the various inflammatory mediators and cytokines, which enhance pulp immune responses against dental pathogens [54].

NALP3 is expressed in human dental pulp cells and in the inflammatory cells and pulp fibroblasts of inflamed pulp, which points to an important role for NALP3 in the recognition of invading pathogens and the initiation of immune responses [44]. The NALP3 inflammasome in pulp fibroblasts is crucial for IL-1*β* secretion in response to LPS, and the latter triggers the TLR4/NF-*κ*B pathway to enhance NALP3 levels in a ROS-dependent manner [55].

Invasion of bacteria or their by-products into the periapical region from an infected root canal system leads to inflammatory reactions that involve various host-derived cells, antibodies, complement and cytokines, and an array of inflammatory mediators, which may cause local tissue destruction in the bone around the periapical tissues, and root resorption. PRR expression is not restricted to macrophages and DCs, in which they have been mainly studied, but includes a variety of cell types, including the gingival fibroblasts that make up the majority of cells in periodontal tissues [84]. Recent research has demonstrated upregulation of TLR1, TLR2, TLR4, and TLR5 at the cell surface and of TLR3, TLR7, TLR8, and TLR9 and NOD1 and NOD2 intracellularly in human gingival fibroblasts [85]. Stimulation of gingival fibroblasts with TLRs and NODs induced the inflammatory cytokines IL-6 and IL-8, an indication that these receptors are active in periodontal tissue. Cementoblasts express TLR4 in response to LPS resulting in alteration of gene expression related to cementum formation, upregulation of osteoclastogenesis-associated molecules such as receptor activator of NF-*κ*B ligand (RANKL) [86]. Stimulation of gingival fibroblasts with TLRs and NODs induced the inflammatory cytokines IL-6 and IL-8, an indication that these receptors are indeed active in periodontal tissue [85]. It is also proved that NALP3 is expressed in the inflammatory periapical tissues [52].

Inflammatory periapical lesions are initiated by polymicrobial infections by Gram-positive and Gram-negative bacteria; they are maintained and exacerbated by prolonged bacterial activity and by their by-products derived from infected root canal systems. Of the various innate and adaptive immune cells found in periapical lesions, most have migrated to the site from the peripheral blood in response to antigens, rather than residing in healthy periapical tissues [45, 81].

## 5. Conclusion

The entry of dental pathogens into dental pulp evokes multiple modes of PRR activation in response to PAMPs. TLRs play a key role in the innate immune system, and ten TLR family members are present in humans (TLR1–TLR10). These differ in their sites of expression and/or ability to recognize different PAMPs. TLRs trigger activation of signaling pathways involving MyD88 and TRIF that lead to the production of proinflammatory cytokines and chemokines via the NF-*κ*B pathway. NLR families include NOD1, NOD2, and NALP3. NLRs are intracellular receptors that recognize PAMPs that have entered the cell and also danger-associated molecular patterns (DAMPs), which are induced during cellular stress. Different levels of NOD1 and NOD2 are activated depending on which pathogenic species is recognized. The ability of PRRs to recognize diverse groups of PAMPs allows the host immune system to respond to encounters with a variety of dental pathogens. Future research needs to clarify the signal transduction pathways subsequent to activation of the PRRs and methods for interfering with PRR activation and their potential therapeutic applications.

## Figures and Tables

**Table 1:** Summary of TLRs and NLRs in human innate immunity.

Family	PRR	Location	Ligand (ligand location)	Unique features
Toll-like receptors (TLRs)	TLR1	Cell surface	Triacyl lipopeptides (bacterial lipoprotein)	Formation of heterophilic dimers with TLR2
	TLR2	Cell surface	Di-/triacyl lipopeptides Multiple lipoproteins Lipoteichoic acid Zymosan (fungi)	Formation of heterophilic dimers with TLR1 and TLR6
	TLR3	Endosome	dsRNA (virus)	dsRNA interacting with the N-terminal and C-terminal sites on the lateral side of convex surface of TLR3
	TLR4	Cell surface	LPS (Gram-negative bacteria)	Recognition of LPS together with myeloid differentiation factor 2
	TLR5	Cell surface	Flagellin	Activation of lung epithelial cells to induce inflammatory cytokine
	TLR6	Cell surface	Triacyl lipopeptides (bacterial lipoprotein)	Formation of heterophilic dimers with TLR2
	TLR7 and TLR8	Endosome	ssRNA (virus)	Recognition of synthetic compound imidazoquinoline
	TLR9	Endosome	Unmethylated CpG DNA	Involvement in the pathogenesis of autoimmune disorders through recognition of the chromatin structure

NOD-like receptors (NLRs)	NOD1	Cytoplasm	Peptidoglycan (Gram-negative bacteria)	Recognition of intracellular bacterial cell products
	NOD2	Cytoplasm	Peptidoglycan (Gram-positive bacteria)	
	NALP3	Endosome	PAMPs, virulence factor DAMPs	Response to multiple stimuli via forming a NALP3 inflammasome

LPS: lipopolysaccharide; NOD: nucleotide-binding oligomerization domain; NALP3: NACHT [neuronal apoptosis inhibitory protein (NAIP), CIITA, HET-E, and TP-1] domain, LRR (leucine-rich repeat) domain, and PYD (pyrin domain) containing protein 3; PAMPs: pathogen-associated molecular patterns; DAMPs: danger-associated molecular patterns.

**Table 2:** Summary of recent finding with PRR in dental pulp.

Author/year/journal	PRR	Cell/animal	Study design	Relevant findings
Durand et al., 2006, J Immunol [32]	TLR	Odontoblast	*In vitro*	LTA upregulates TLR2 and chemokine expression while downregulating dentin matrix synthesis and mineralization

Jiang et al. 2006 J Endod [33]	TLR4	Odontoblast Dental pulp tissue	*In vitro*	TLR4 expression in normal DP

Mutoh et al., 2007, J Endod [34]	TLR2, TLR4	Murine pulp tissue	*In vivo*	TLR2, TLR4 expression in DP TLR2 regulates early stage of pulp inflammation

Marcato et al., 2008, Oral Microbiol Immunol [35]	TLR2, TLR4	Mouse model	*In vivo*	TLR2, TLR4 induce NO and ROS production by macrophage stimulated with root canal pathogens

Mutoh et al., 2009, J Endod [36]	TLR2, TLR4	SCID mice	*In vivo*	TLR2, TLR4 are triggered by dental pathogen in irreversible pulpitis

Horst et al., 2009, J Dent Res [37]	TLR2, TLR4	Odontoblast	*In vitro*	TGF-*β*1 inhibits TLR2, TLR4 expression against dental pathogens

Hirao et al., 2009, J Dent Res [16]	TLR2, TLR4 and NOD1, NOD2	HDPF	*In vitro*	TLR2, TLR4, NOD1, and NOD2 expression in DP NOD2 is an immunomodulator through TLR2, leading to progressive pulpitis

Lin et al., 2009, J Endod [38]	NOD2	HDPC	*In vitro*	NOD2 expression in normal DP

Keller et al., 2010, Immunobiology [39]	TLR2	Odontoblast HDPF	*In vitro*	LTA upregulates TLR2 in odontoblasts and HDPF

Park et al., 2010, J Dent Res [40]	TLR2	HDPF	*In vitro*	TLR2 on HDPF with histamine receptor-1 induces pulpal inflammation via Cox-2 activation

Botero et al., 2010, J Dent Res [41]	TLR4	HDPSC (HDPF)	*In vitro*	LPS upregulates VEGF DPSC express TLR4

Farges et al., 2011, Immunobiology [42]	TLR2	Odontoblast	*In vitro*	TLR2 engages production of mediators in odontoblasts

Keller et al., 2010, Innate Immun [43]	TLR2, NOD2	HDPC, odontoblast	*In vitro*	Upregulation of TLR2, NOD2 through stimulation via LTA in inflamed DP

Lee et al., 2011, J Endod [18]	NOD1	HDPF	*In vitro*	Upregulation of NOD1 in inflamed DP

Song et al., 2012, J Endod [44]	NALP3	HDPF	*In vitro*	NALP3 upregulates in dental pulp immune defense

Da Silva et al., 2012, J Endod [45]	TLR2	TLR2 KO mice	*In vivo*	TLR2 regulates inflammatory response and host's immune to root canal and periradicular infection

Carrouel et al., 2013, J Endod [46]	TLR2	Odontoblast	*In vitro*	LBP reduces TLR2-dependant immune responses by LTA in human odontoblast-like cells

Zhang et al., 2013, Int Endod J [47]	TLR9	Odontoblast	*In vitro*	TLR9 regulates the remodeling of injured DP and hard tissues by inducing MMP-13

He et al., 2013, Int Endod J [5]	TLR4	HDPSC	*In vitro*	LPS upregulates IL-8 with engagement of TLR4/MyD88/NF-κB and MAPK pathways in DP

Keller et al., 2011, Innate Immun [43]	NOD2	Odontoblast	*In vitro*	LTA augmented NOD2 expression in odontoblasts

Wang et al., 2013, J Endod [48]	AIM2	Rat model Rat pulp cell	*In vivo* *In vitro*	AIM2 is only detected in the odontoblast layer and mediates inflammatory response during pulpitis

Cardoso et al., 2014 J Endod [49]	TLR2	Inflamed and healthy human dental pulp tissue	*In vitro*	Hypomethylation of TLR2 and CD14 gene mediates immune responses against LPS

He et al., 2014 J Endod [50]	TLR4	HDPSC	*In vitro*	LPS enhances Wnt5a expression via TLR4/MyD88/NF-κB pathways in DP

Feng et al., 2014, Cell Tissue Res [51]	TLR4	HDPSC	*In vitro*	LPS+ TLR4 complex stimulates inflammation in DP

Liu et al., 2014, J Endod [52]	TLR4	HDPSC	*In vitro*	LPS activates TLR4 TLR4 regulates the proliferation and migration of DPSC in deep dental caries

Pääkkönen et al., 2014, Int Endod J [53]	TLR3, TLR7, TLR8, and TLR9	Odontoblast	*In vitro*	TLR3, TLR7, TLR8, and TLR9 mRNA (virus recognition PRR) participate in immune response in DP

Lee et al., 2014, Clin Oral Invest [54]	TLR2, TLR4, and NALP3	HDPC	*In vitro*	TLR and NALP3 activate immune responses during progression of pulpitis

Zhang et al., 2015, Mol Immunol [55]	NALP3, TLR4	HDPF	*In vitro*	NALP3 in HDPFs triggers IL-1 secretion in response to LPS plus ATP LPS engaged TLR4/MyD88/NF-κB pathway to enhance NLRP3

Lee et al., 2014, Clin Oral Invest [54]	NALP3	HDPF	*In vitro*	NOD2 activates TLR2, TLR4, and NALP3 inflammasome-signaling pathways

Liu et al., 2014, Int Endod J [52]	NALP3	HDPSC	*In vitro*	NALP3 expressed in periapical lesion

TLR: Toll-like receptor; LTA: lipoteichoic acid; DP: dental pulp; NO: nitric oxide; ROS: reactive oxygen species; SCID: severe combined immunodeficiency mice; TGF-*β*1: transforming growth factor-*β*1; HDPF: human dental pulp fibroblast; HDP(S)C: human dental pulp (stem) cell; NOD: nucleotide-binding oligomerization domain; Cox: cyclooxygenase; LPS: lipopolysaccharide; VEGF: vascular endothelial growth factor; DPSC: dental pulp stem cell; NALP: NACHT [neuronal apoptosis inhibitory protein (NAIP), CIITA, HET-E, and TP-1]; KO: knockout; LBP: lipopolysaccharide-binding protein; MMP: matrix metalloproteinase; MyD88: myeloid differentiation factor 88; NF-κB: nuclear factor kappa B; MAPK: mitogen-activated protein kinase; AIM: absent in melanoma; ATP: adenosine triphosphate.
